# Preclinical Mechanistic Evaluation of Hyaluronan/Niacinamide (Vitamin B3) Hydrogels: Toward an Enhanced Viscosupplement System with Ancillary Anti-Arthritic Attributes

**DOI:** 10.3390/bioengineering12111246

**Published:** 2025-11-14

**Authors:** Farid Hadjab, Stivens Antoine, Béatrice Hamel, Mohamed Benderdour, Hassan Fahmi, Alexandre Porcello, Virginie Philippe, Robin Martin, Cíntia Marques, Kelly Lourenço, Corinne Scaletta, Nathalie Hirt-Burri, Philippe Abdel-Sayed, Lee Ann Applegate, Alexis E. Laurent

**Affiliations:** 1 Development Department, Albomed GmbH, D-90592 Schwarzenbruck, Germany; f.hadjab@albomed.eu; 2 Laboratoire de Recherche en Orthopédie, Centre de Biomédecine, Hôpital du Sacré-Cœur de Montréal, Montreal, QC H4J 1C5, Canada; stivens.antoine@outlook.com (S.A.); beatrice.hamel@umontreal.ca (B.H.); mohamed.benderdour@umontreal.ca (M.B.); 3 Osteoarthritis Research Unit, University of Montreal Hospital Research Center (CRCHUM), Montreal, QC H2X 0A9, Canada; h.fahmi@umontreal.ca; 4 Development Department, LOUNA REGENERATIVE SA, CH-1207 Geneva, Switzerland; a.porcello@louna-aesthetics.com (A.P.); c.marques@louna-aesthetics.com (C.M.); k.lourenco@louna-aesthetics.com (K.L.); 5 Regenerative Therapy Unit, Lausanne University Hospital, University of Lausanne, CH-1066 Epalinges, Switzerland; virginie.philippe@chuv.ch (V.P.); corinne.scaletta@chuv.ch (C.S.); nathalie.burri@chuv.ch (N.H.-B.); philippe.abdel-sayed@chuv.ch (P.A.-S.); lee.laurent-applegate@chuv.ch (L.A.A.); 6 Orthopedics and Traumatology Unit, Lausanne University Hospital, University of Lausanne, CH-1011 Lausanne, Switzerland; robin.martin@chuv.ch; 7 STI School of Engineering, Federal Polytechnic School of Lausanne, CH-1015 Lausanne, Switzerland; 8 Center for Applied Biotechnology and Molecular Medicine, University of Zurich, CH-8057 Zurich, Switzerland; 9 Oxford OSCAR Suzhou Center, Oxford University, Suzhou 215123, China; 10 Manufacturing Department, TEC-PHARMA SA, CH-1038 Bercher, Switzerland; 11 Manufacturing Department, LAM Biotechnologies SA, CH-1066 Epalinges, Switzerland

**Keywords:** antioxidants, biophysical properties, degradation, hyaluronic acid, hydrogels, inflammation, niacinamide, osteoarthritis, primary chondrocytes, viscosupplements

## Abstract

Osteoarthritis (OA), a degenerative joint disease primarily affecting the hips and knees, is characterized by multifactorial dysregulation of chondrocyte homeostasis and currently lacks curative treatment options. Intra-articular hyaluronic acid (HA) injections have clinically provided symptomatic relief for three decades; however, HA’s rapid in vivo degradation by free radicals and hyaluronidases limits its efficacy. We hypothesized that adding niacinamide (vitamin B3) to linear HA hydrogels would provide ancillary anti-inflammatory and anti-catabolic properties, thereby improving HA-based viscosupplementation therapy. This preliminary preclinical mechanistic study investigated the functional effects of incorporating niacinamide into linear HA-based hydrogels using in vitro cellular models. Initially, Raw 264.7 macrophages and C28/I2 or SW1353 human chondrocytes were pre-treated with varying concentrations of HA/B3, with or without lipopolysaccharide (LPS) or interleukin-1β (IL-1β), respectively. Subsequently, pro-inflammatory and pro-catabolic markers were quantified biochemically. Results demonstrated that HA/B3 hydrogels exhibited enhanced functional stability compared to HA alone and possessed significant anti-inflammatory and anti-catabolic properties, without inducing cytotoxicity in either cell line. In Raw 264.7 macrophages, HA/B3 inhibited LPS-induced tumor necrosis factor-α (TNF-α) release and suppressed cyclooxygenase-2 (COX-2) and inducible nitric oxide synthase (iNOS) protein expression. In vitro, HA/B3 hydrogels reduced IL-1β-induced IL-6 production in primary chondrocytes by 16% and suppressed PGE_2_ concentration in both macrophages and chondrocytes by 60%, effects superior to HA alone. Finally, a rat primary articular chondrocyte model suggested slight anti-hypertrophic effects of HA/B3 in vitro. Collectively, these findings suggest that HA/B3 hydrogels possess anti-arthritic potential, highlighting a novel strategy for next-generation viscosupplement systems.

## 1. Introduction

Osteoarthritis (OA) stands as one of the most prevalent degenerative joint diseases, characterized by the progressive deterioration of articular cartilage, ultimately leading to subchondral bone exposure. This pathological process predominantly affects the knees and hip joints, significantly impairing patient mobility and quality of life. While aging generally contributes to joint degeneration, OA arises from a complex disruption of the homeostatic equilibrium within the affected joints [1,2]. Notably, the onset and progression of OA are associated with a progressive decline in the local concentration of critical macromolecules within the chondral extracellular matrix (ECM) [1,3,4]. This imbalance manifests as a disparity between the synthesis and degradation of cartilage-specific ECM components, particularly hyaluronic acid (HA) and type II collagen. The loss of these macromolecules is largely attributed to dysregulated chondrocyte metabolism, mediated by pro-inflammatory cytokines such as interleukin-1β (IL-1β) and tumor necrosis factor-α (TNF-α) [3,5]. These cytokines not only suppress the synthesis of essential cartilage-maintaining macromolecules but also promote their degradation by stimulating the production of catabolic enzymes [6,7,8,9]. Other additional factors, such as reactive oxygen species (ROS) and lipid peroxidation products, play an important role in the regulation of these processes in OA [10,11].

Cartilage, a specialized connective tissue, comprises a sparse population of chondrocytes embedded within an ECM rich in type II collagen, hyaluronan, and aggrecan. At the cellular level, IL-1β triggers the activation of matrix-degrading enzymes and suppresses the expression of matrix components, including type II collagen and aggrecan. Chondrocytes, the sole resident cells in articular cartilage, play a pivotal role in OA development by regulating ECM production [5,12]. In healthy cartilage, collagens, HA, glycoproteins, and proteoglycans provide a smooth, lubricated joint surface capable of compression, stretching, and frictionless gliding [3]. Enzymatic degradation mediated by matrix metalloproteinases (MMPs) and aggrecanases, together with oxidative stress, progressively alters these structural and biochemical components and weakens the extracellular matrix. Consequently, any disruption in the structure and composition of hyaline cartilage surfaces can precipitate clinically evident signs of OA.

Despite ongoing clinical research, OA remains incurable, with surgical joint replacement as the eventual solution. Current management strategies focus on pain alleviation and joint function preservation [13,14]. Intra-articular HA injections, or viscosupplementation, are widely used for symptomatic relief in order to provide an additional cushioning effect and lubrication to reduce pain and stiffness and to improve motion. However, despite its favorable biological and physicochemical properties, exogenous HA is rapidly degraded in vivo by free radicals and hyaluronidases, limiting its therapeutic efficacy [15,16,17]. Several studies have explored strategies to improve the stability and persistence of HA in biological environments, particularly by incorporating antioxidant carbohydrates such as mannitol and sorbitol. Mannitol is a polyol well known for its properties as a ROS scavenger. Its beneficial antioxidant effects are largely attributed to the abundance of reactive hydroxyl groups, which can neutralize free radicals and prevent oxidative chain scission of HA [18,19,20]. Such stabilization approaches aim to prolong HA’s biological activity, preserve its viscoelastic and lubricating properties, and maintain its structural integrity during mechanical loading. Additionally, commercial HA-based viscosupplementation products are available that include carbohydrate stabilizers such as sorbitol and mannitol. For instance, Synolis VA (Aptissen, Plan-les-Ouates, Switzerland) contains 80 mg of sorbitol, while Ostenil^®^ Plus (TRB Chemedica, Carouge, Switzerland) includes 10 mg of mannitol. Of note, although platelet-rich plasma (PRP) and stem cell injections demonstrate superior efficacy, intra-articular HA remains the most common approach due to its global affordability, ease of administration, and excellent tolerability [21,22]. Intriguingly, despite its long-standing use in orthopedics, the precise mechanisms of HA’s therapeutic action in OA are not yet fully elucidated [23].

HA, even in small quantities, imparts significant viscosity, elasticity, lubrication, and molecular segregation. Intra-articular HA injections in OA patients help maintain the physiological characteristics (viscosity, hydration, elasticity, and pH) of the endogenous ECM and synovial fluid [23,24,25]. HA participates in diverse biological processes, including cellular proliferation, differentiation, mobility, and intercellular interactions, as well as the modulation of inflammatory mediators. Notably, the effects of HA-based hydrogels are dependent on the molecular weight distribution of the HA polymer [24,26,27,28]. Low molecular weight HA molecules (i.e., below 700 kDa) exhibit pro-inflammatory and catabolic properties, whereas high-molecular-weight HA (i.e., over 1000 kDa) polymers possess anti-inflammatory, antioxidant, and analgesic effects [28].

The intricate metabolic pathways of exogenous HA remain incompletely understood. Recent findings suggest that high molecular weight HA primarily acts through intercellular adhesion molecule 1 (ICAM-1), hyaluronan-mediated motility receptor (i.e., HMMR; RHAMM, CD168), and the CD44 cluster determinant [29,30,31]. In articular chondrocytes, HA binding to its membrane receptors, with or without HA-binding proteins, triggers cytoplasmic signaling cascades and transcriptional effects via CD44 and hyaluronan-binding protein 2 (HYAL-2) endocytosis and nuclear translocation [24,31,32,33,34,35,36]. Specifically, HA-mediated signaling through CD44 and RHAMM influences cellular proliferation, differentiation, cytokine production, as well as HA and collagen synthesis [31,34,37].

This in vitro preclinical study aimed to demonstrate a novel strategy for enhancing HA hydrogels’ anti-arthritic attributes by incorporating niacinamide, a form of vitamin B3. The rationale for niacinamide addition is based on its intrinsic antioxidant and anti-inflammatory properties, as well as its demonstrated beneficial effects in OA [38,39]. In our previous study, the stability of the HA/B3 combination was evaluated and compared to commercial HA viscosupplement hydrogels in terms of rheological behavior, lubricating performance, and adhesive properties (i.e., only mechanical evaluations were performed). Subsequently, its antioxidant capacity (ROS reduction and antioxidant enzyme activity enhancement) was investigated. The incorporation of small-molecule antioxidants such as niacinamide likely enhances non-ionic and hydrogen-bonding interactions within the HA network, promoting a more stable conformation and greater resistance to oxidative degradation [38]. These interactions may help preserve the viscoelastic and lubricating properties of HA under oxidative stress. However, the precise mechanism by which vitamin B3 stabilizes HA under oxidative conditions remains to be fully elucidated. Further, its anti-inflammatory, anti-catabolic, and anabolic effects were explored using immortalized human chondrocytes and macrophages treated with varying doses of HA/B3, with or without IL-1β or lipopolysaccharide (LPS) induction. Inflammatory, oxidative stress, catabolic, and signaling pathway biomarkers were assessed. Finally, the anti-hypertrophic effects (i.e., type II collagen and aggrecan expression) of the HA/B3 combination were examined in a primary rat chondrocyte model. This study sought to validate the hypothesis that niacinamide incorporation into HA hydrogels confers multiple functional benefits, paving the way for novel injectable therapeutic options for OA.

## 2. Materials and Methods

### 2.1. Investigational Sample Preparation

For in vitro assays utilizing cell lines, high molecular weight sodium hyaluronate powder (>2.0 MDa; samples were provided by Albomed, Schwarzenbruck, Germany) was sterilized using steam sterilization and subsequently dissolved in Dulbecco’s Modified Eagle Medium (DMEM; Wisent Bioproducts, Saint-Jean-Baptiste, QC, Canada) supplemented with 10% *v*/*v* fetal bovine serum (FBS; Wisent Bioproducts, QC, Canada), 100 units/mL penicillin, and 100 μg/mL streptomycin. Niacinamide powder (samples provided by Albomed) was also dissolved in DMEM (Wisent Bioproducts, QC, Canada) supplemented with 10% *v*/*v* FBS (Wisent Bioproducts, QC, Canada), 100 units/mL penicillin, and 100 μg/mL streptomycin. Both solutions were then sterile-filtered using 0.22 μm membrane filters. The HA and niacinamide (B3) solutions were simply mixed (i.e., hydration for 3 h followed by low-speed planetary mixing for 4 h at room temperature) to achieve final concentrations of 2.00 mg/mL and 1.33 mg/mL, respectively, and stored protected from light at 4 °C until use. For in vitro assays employing rat primary chondrocytes, sterile hydrogels containing 0.25% HA, with or without 2.5% niacinamide, were provided by Albomed (Schwarzenbruck, Germany). These samples were stored protected from light at 4 °C until use.

### 2.2. Cell Lines and Primary Chondrocyte Cell Type

Raw 264.7 cells (ATCC, Manassas, VA, USA), SW1353 cells (ATCC), and C28/I2 (kindly provided by Dr M. Goldring) (passage level ≤ 12) were cultured in 24-well plates using Dulbecco’s Modified Eagle Medium (DMEM; Wisent Bioproducts, QC, Canada) supplemented with 10% *v*/*v* FBS (Wisent Bioproducts, QC, Canada), 100 units/mL penicillin, and 100 μg/mL streptomycin. Cultures were maintained at 37 °C in a humidified atmosphere with 5% CO_2_ for 24 h, allowing cells to reach approximately 85% confluency prior to treatment. The culture medium was replaced with fresh medium before the addition of test compounds. Cells from each cryovial were maintained in culture for a maximum period of 4 weeks.

Primary rat chondrocytes were isolated from the knee cartilage of 3-week-old Sprague Dawley rats. Briefly, knee cartilage was aseptically dissected from the subchondral bone using a sterile scalpel. Cartilage shavings were digested with 1 mg/mL type II collagenase in serum-free DMEM (Biowest, Nuaillé, France). Following complete tissue digestion, the cell suspension was centrifuged to obtain a cell pellet. This pellet was then resuspended in DMEM containing 10% *v*/*v* FBS, and the cells were counted. The isolated chondrocytes were plated in plastic tissue culture vessels at a density of 10^4^ cells/cm^2^. These primary chondrocytes were expanded as a monolayer using proliferation medium (DMEM/10% FBS, supplemented with 25 mM HEPES) until passage 1 and subsequently cryopreserved at −80 °C until required for experimentation.

### 2.3. MTS Cellular Viability Assays

Cellular viability in response to varying concentrations in µg/mL of hyaluronic acid (HA), niacinamide, and the HA/niacinamide (HA/B3) combination was assessed using the MTS assay (3-(4,5-dimethylthiazol-2-yl)-5-(3-carboxymethoxyphenyl)-2-(4-sulfophenyl)-2H-tetrazolium; Promega, Madison, WI, USA). Raw 264.7 and C28 cell lines were seeded in 96-well plates at densities of 3.5 × 10^4^ cells/well and 3.0 × 10^4^ cells/well, respectively. Cells were incubated for 24 h in DMEM (Wisent Bioproducts, QC, Canada) supplemented with 10% *v*/*v* FBS (Wisent Bioproducts, QC, Canada), 100 units/mL penicillin, and 100 μg/mL streptomycin, maintained at 37 °C in a humidified atmosphere with 5% CO_2_.

Following the 24 h incubation, the culture medium was replaced with fresh medium containing the specified treatments: HA at 0, 10, 20, 50, 100, 250, and 500 μg/mL; niacinamide at 0, 6.6, 13.3, 66.6, 166.6, and 333.3 μg/mL; and HA/B3 combinations at the corresponding respective concentrations. Plates were then incubated for an additional 24 h under the same conditions.

After this 24 h exposure period, the MTS assay was performed according to the manufacturer’s instructions. Absorbance readings were obtained at 490 nm using a multi-well plate reader (EL800, Bio-Tek Instruments, Winooski, VT, USA). Hydrogen peroxide (1000 μM for Raw 264.7 cells and 750 μM for SW1353 cells) was used as a positive control for cytotoxicity. Given the absence of a defined viability limit in the MTS assay according to ISO 10993-5, a viability rate below 80% was designated as indicative of a toxic effect in this study [40].

### 2.4. Modulation of Protein Synthesis in Cell Lines

Both Raw 264.7 and C28/I2 cell lines were pre-treated with increasing concentrations of hyaluronic acid (HA; 0, 10, 20, 50, 100, 250 μg/mL), niacinamide (0, 6.6, 13.3, 66.6, 166.6 μg/mL), and HA/niacinamide (HA/B3) combinations at the corresponding concentrations. Cells were incubated for 2 h at 37 °C in a humidified atmosphere with 5% CO_2_. Subsequently, cells were incubated for 24 h under the same conditions, in the presence or absence of either a free radical generator or an inflammatory agent relevant to osteoarthritis (OA) pathogenesis: 50 ng/mL lipopolysaccharide (LPS) for the macrophage cell line and 1 ng/mL interleukin-1β (IL-1β) for the chondrocyte cell line.

Following the 24 h incubation period, the culture medium was collected, and cells were lysed using Laemmli lysis buffer (Bio-Rad, Hercules, CA, USA; 300 μL for Raw 264.7 cells and 200 μL for C28 cells). Cell lysates were incubated at 96 °C for 5 to 10 min and subsequently stored at −20 °C for short-term storage or −80 °C for long-term storage until further analysis.

### 2.5. Modulation of Signaling Pathways in Cell Lines

Both Raw 264.7 and C28/I2 cell lines were pre-treated with increasing concentrations of the hyaluronic acid (HA) formulation (0, 10, 20, 50, 100, 250 μg/mL) and niacinamide (0, 6.6, 13.3, 66.6, 166.6 μg/mL) or combinations thereof. Cells were incubated for 2 h at 37 °C in a humidified atmosphere with 5% CO_2_. Subsequently, cells were incubated for 1 h under the same conditions, in the presence or absence of either a free radical generator or inflammatory agents relevant to osteoarthritis (OA) pathogenesis: 50 ng/mL lipopolysaccharide (LPS) for the macrophage cell line and 1 ng/mL interleukin-1β (IL-1β) for the chondrocyte cell line. Following the 1 h incubation period, the culture medium was removed, and cells were lysed using Laemmli lysis buffer (Bio-Rad, Hercules, CA, USA; 300 μL for Raw 264.7 cells and 200 μL for C28 cells). Cell lysates were incubated at 96 °C for 5 to 10 min and subsequently stored at −20 °C for short-term storage or −80 °C for long-term storage until further analysis.

### 2.6. ELISA Dosing of PGE_2_, TNF-α, IL-1β, and MMP-13 in Cell Lines

The culture medium collected after 24 h of incubation with macrophages and chondrocytes was analyzed using enzyme-linked immunosorbent assays (ELISAs, Elisa, Finland) according to the manufacturer’s protocols provided with each kit. Specifically, the culture medium from macrophage treatments was assayed for prostaglandin E_2_ (PGE_2_) using a monoclonal prostaglandin E_2_ ELISA kit (Cayman Chemical, Ann Arbor, MI, USA), tumor necrosis factor-alpha (TNF-α) using a mouse TNF-α/uncoated ELISA kit (Invitrogen, Carlsbad, CA, USA), and interleukin-1β (IL-1β) using a mouse IL-1β uncoated ELISA kit (Invitrogen). Similarly, the culture medium from chondrocyte treatments was analyzed for PGE_2_ using a monoclonal prostaglandin E_2_ ELISA kit (Cayman Chemical) and matrix metalloproteinase-13 (MMP-13) using a human total MMP-13 DuoSet ELISA kit (R&D Systems, Minneapolis, MN, USA).

### 2.7. Dosing of Nitric Oxide

Nitric oxide (NO) levels were quantified using a modified NCL method ITA-7 [41]. Culture medium collected after 24 h of incubation with macrophages and chondrocytes was added to a 96-well plate (50 μL/well). Griess reagent (100 μL) was subsequently added, and the plate was incubated for 15 to 30 min, protected from light. Absorbance was measured at 550 nm. A calibration curve was generated using sodium nitrite solutions at concentrations of 250, 125, 62.5, 31.3, 15.6, 7.8, 3.91, and 1.95 μM. DMEM (Wisent Bioproducts, QC, Canada) supplemented with 10% *v*/*v* FBS (Wisent Bioproducts, QC, Canada), 100 units/mL penicillin, and 100 μg/mL streptomycin was used as a negative control and as a diluent for preparing the sodium nitrite calibration solutions.

### 2.8. Western Blot Detection of COX-2, iNOS, P38, NF-κB, and ERK1/2

Cell lysates were incubated at 96 °C for 5 to 10 min and subsequently loaded (15 μL) onto sodium dodecyl sulfate–polyacrylamide gels (SDS-PAGE; 4.0–7.5% gel for COX-2 and iNOS, 4–10% gel for p38, NF-κB, and ERK1/2). Proteins were separated by electrophoresis at 120 V for 75 min. Separated proteins were then transferred to nitrocellulose membranes (Bio-Rad Laboratories, Mississauga, ON, Canada) for immunodetection and semi-quantitative analysis. Primary antibodies specific to COX-2 (Cayman Chemicals), iNOS (Cayman Chemicals), p38 (Cell Signaling Technology, Danvers, MA, USA), NF-κB/p65 (Cell Signaling Technology), and ERK1/2 (Cell Signaling Technology) were used for protein detection. Proteins were visualized using a peroxidase-conjugated secondary antibody (Cell Signaling Technology), in conjunction with its substrate (Clarity Western ECL substrate, Bio-Rad), and exposed to Kodak X-Omat film (Eastman Kodak Company, Rochester, NY, USA). For mitogen-activated protein kinases (MAPKs; p38, NF-κB, and ERK1/2), both the total enzyme and its phosphorylated (active) form were measured.

### 2.9. Evaluation of Anti-Hypertrophic Effects on Rat Chondrocytes

#### 2.9.1. In Vitro Assay Design

The effects of HA/B3 on primary chondrocytes treated with IL-1β (i.e., 10 ng/mL) were assessed. The modification of the expression of type II collagen, aggrecan, and MMP-13 was investigated by qPCR analyses. Moreover, the levels of two markers of inflammation (i.e., IL-6 and PGE_2_) were measured in the culture supernatants. Thawed chondrocytes were seeded and cultured in monolayers in 12-well plates for 24 h. Then, the cultures were treated with IL-1β and with the various test compounds for three days. The following five treatments or control conditions were experimentally used:

- Untreated cells (i.e., cultures in proliferation medium);
- IL-1β-treated cells;
- IL-1β-treated cells + 1:10 dilution of HA sample without niacinamide;
- IL-1β-treated cells + 1:10 dilution of HA sample with niacinamide;
- IL-1β-treated cells + 1:100 dilution of HA sample with niacinamide.

All treatments and controls were carried out in triplicate. The endpoint readouts performed included RT-qPCR analyses for the expression of collagen II, aggrecan, and MMP-13, as well as ELISA quantifications for IL-6 and PGE_2_ production.

#### 2.9.2. Preparation of Test Compounds

Culture medium supplemented with 20 ng/mL of IL-1β was prepared with recombinant rat IL-1β. Working solutions of the test compounds were diluted 1:2 in proliferation medium supplemented with 20 ng/mL of IL-1β, in order to treat the cells with 10 ng/mL of IL-1β and with 1:10 or 1:100 dilutions of the test compounds.

#### 2.9.3. qPCR Analyses

Following 72 h of treatment, chondrocytes were harvested, lysed, and total RNA was purified using the NucleoSpin RNA II kit (Macherey Nagel, Düren, Germany). Total RNA was reverse-transcribed using the M-MLV RT reagent (Life Technologies, Carlsbad, CA, USA). The resulting cDNA strands were used as templates for qPCR to determine the steady-state mRNA levels of type II collagen, aggrecan, and MMP-13.

To normalize the qPCR results, the steady-state mRNA levels of two housekeeping genes, ribosomal protein L19 (RPL19) and β-actin, were also determined and used as internal loading controls. Each qPCR reaction was prepared as follows: 5 μL of iTaq Universal SYBR Green Supermix (Bio-Rad), 0.6 μL of forward primer (5 μM), 0.6 μL of reverse primer (5 μM), 1.8 μL of H_2_O, and 2 μL of cDNA. The sequences of the forward and reverse primers that were used are detailed in Table 1.

All qPCR reactions were performed using a CFX Connect Real-Time PCR Detection System (Bio-Rad). Each qPCR experiment was conducted in triplicate. A total of 36 qPCR reactions were performed per marker, encompassing four treatments or control conditions, each analyzed in triplicate, with each qPCR reaction itself also performed in triplicate. The CFX software V 2.3 was utilized for qPCR data analysis.

The mean cycle threshold (Ct) value was calculated from the technical triplicates for each marker and the two housekeeping genes. From these raw Ct values, the expression ratio between test and reference samples was determined. Test and reference samples were manually designated.

To calculate the expression ratio from Ct values, the software determined (1) the relative mRNA quantity for the target gene and the two reference genes; (2) the relative mRNA quantity of the target gene normalized to the reference genes; and (3) the expression ratio, which represents the normalized relative quantity of the target gene in a test sample divided by the normalized relative quantity of the target gene in a reference sample.

#### 2.9.4. ELISA Analyses

Following 72 h of treatment, culture supernatants were collected, aliquoted, and stored at −20 °C until further analysis. The quantification of IL-6 and PGE_2_ was performed according to the protocols provided by the suppliers of the respective enzyme-linked immunosorbent assay (ELISA) kits: rat IL-6 Quantikine ELISA kit (R&D Systems) and PGE_2_ ELISA kit (Enzo Life Sciences, Farmingdale, NY, USA). Absorbance measurements were obtained using a Multiskan Go spectrophotometer plate reader. The SkanIt 3.2 software was employed to perform calculations on the measurement data, specifically to determine concentrations from optical density (OD) values. Statistical significance between test samples and the IL-1β-treated samples was determined using the Mann–Whitney U test.

### 2.10. Statistical Analyses and Data Presentation

Experimental data are presented as mean values with corresponding standard deviations, which are graphically represented as error bars. For comparisons across multiple groups, statistical analyses were performed using either one-way analysis of variance (ANOVA) followed by Tukey’s multiple comparison test or the Mann–Whitney U test. A *p*-value of less than 0.05 was considered the threshold for statistical significance. Detailed levels of statistical significance are specified in the Results section. Statistical calculations and data visualization were performed using Microsoft Excel 365 (Microsoft Corporation, Redmond, WA, USA), Microsoft PowerPoint 365, and GraphPad Prism version 8.0.2 (GraphPad Software, San Diego, CA, USA).

## 3. Results and Discussion

### 3.1. Cellular Viability Evaluation by MTS Assays

**Figure 1:**
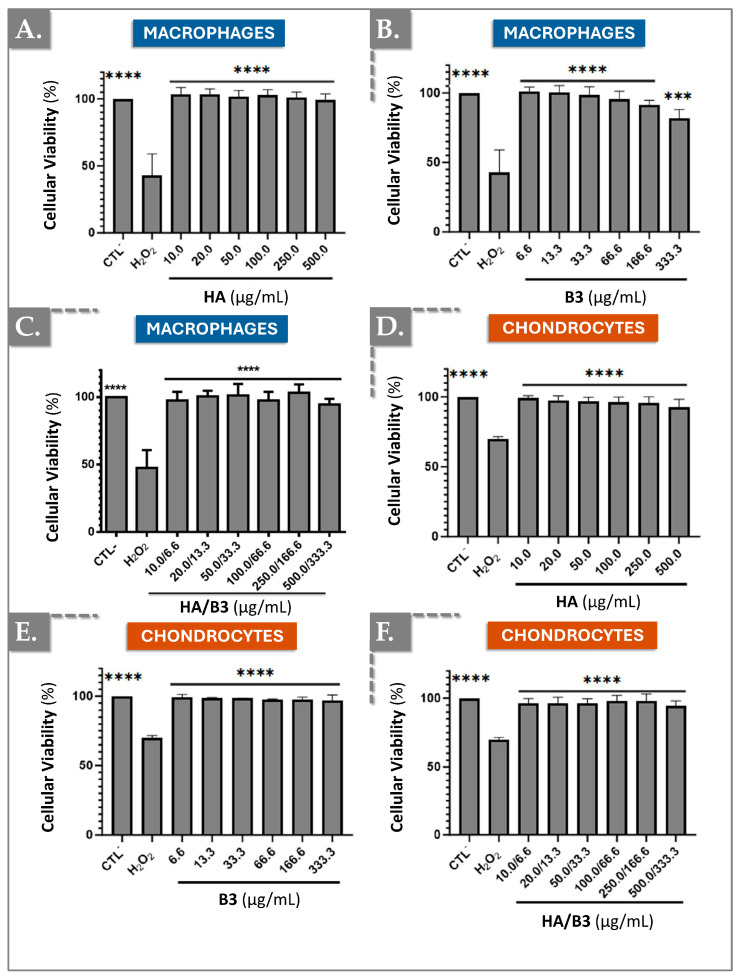
Investigation of potential cytotoxic effects of HA, B3, or HA/B3 combinations on Raw 264.7 macrophages (**A**–**C**) and SW1353 chondrocytes (**D**–**F**). The cells were incubated for 24 h at 37 °C with different concentrations of test compounds. The MTS assay was performed at the endpoint. Untreated cells and hydrogen peroxide-treated cells (i.e., 1000 μM for Raw 264.7 and 750 μM for SW1353 cells) were used as controls. Statistically significant differences (i.e., **** or *p*-value < 0.0001; *** or *p*-value < 0.001) were outlined between the groups. Data are expressed as the mean of three independent tests (N = 3) and analyzed by one-way ANOVA (i.e., with a post hoc Tukey test). HA, hyaluronic acid.

To evaluate the potential cytotoxic effects of hyaluronic acid (HA), niacinamide (B3), and their combined formulation on cell lines, an MTS-based cellular viability assay was performed. This assay quantifies cellular viability by measuring the reduction in tetrazolium to formazan, a soluble product that can be spectrophotometrically detected at 490–500 nm. Specifically, this reduction is mediated by NAD(P)H-dependent dehydrogenase enzymes present in metabolically active, viable cells. In this study, Raw 264.7 macrophages and SW1353 chondrocytes were directly exposed to varying concentrations of HA (i.e., from 10.0 µg/mL to 500.0 µg/mL), B3 (i.e., from 6.6 µg/mL to 333.3 µg/mL), and HA/B3 (i.e., from 10.0/6.6 µg/mL to 500.0/333.3 µg/mL) combinations for 24 h, after which cellular viability was determined. Untreated cells served as a negative control, while hydrogen peroxide (H_2_O_2_)-treated cells (i.e., 50 µg/mL) were used as a positive control for cytotoxicity. As depicted in Figure 1, HA exposure did not significantly impact the viability of either cell line.

Notably, niacinamide exhibited a more pronounced effect on Raw 264.7 macrophages, where cellular viability demonstrated a progressive decline with increasing niacinamide concentrations (Figure 1B). This observation was not replicated in SW1353 chondrocytes (Figure 1E). A similar trend, though less pronounced, was observed in cells exposed to the HA/B3 combination (Figure 1C,F). However, the variations in viability were not statistically significant compared to untreated cells. These findings suggest that, at the tested concentrations, HA, B3, and their combined formulation elicited minimal cytotoxic effects on both cell lines. Nevertheless, niacinamide appeared to induce a discernible reduction in viability at higher concentrations in Raw 264.7 macrophages (Figure 1).

### 3.2. The HA/B3 Combination Inhibits NO and PGE_2_ Production by Reducing iNOS and COX-2 Expression

**Figure 2:**
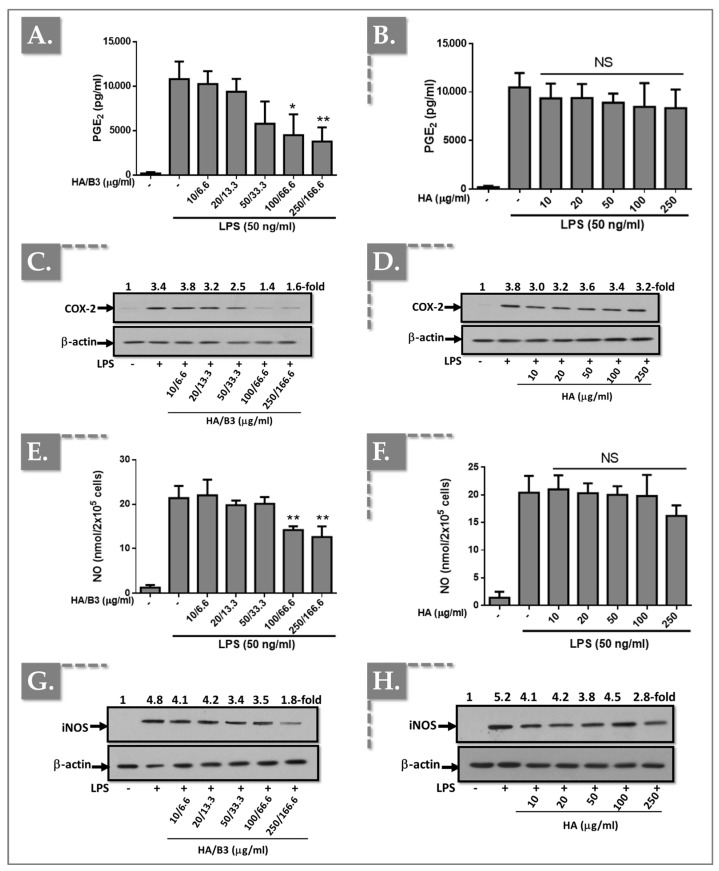
Investigation of the anti-inflammatory effects of the HA/B3 combination in Raw 264.7 macrophages. The cells were pretreated with increasing concentrations of HA alone or HA/B3 for 2 h. After this period, they were induced with 50 ng/mL LPS. They were then incubated for 24 h at 37 °C under 5% CO_2_ in a humidified environment. Then, 50 μL of culture medium was isolated and used for PGE_2_ (**A**,**B**) and NO assays (**E**,**F**). Samples were diluted 20-fold for the PGE_2_ assay. Volumes of 15 μL of cell lysate were loaded onto SDS-PAGE polyacrylamide gels, followed by transfer to nitrocellulose membranes. COX-2 (**C**,**D**) and iNOS (**G**,**H**) proteins were revealed by immunoblotting and detected with Rochester photosensitive film. Statistically significant differences (i.e., ** or *p*-value < 0.01; * or *p*-value < 0.05) are outlined between the groups. Data are expressed as the mean of three independent tests (N = 3) and analyzed by one-way ANOVA (i.e., with a post hoc Tukey test). HA, hyaluronic acid; LPS, lipopolysaccharide; NO, nitric oxide. - not added, + treated, NS Not Significant.

To investigate the effects of the HA/B3 formulation on pro-inflammatory enzyme expression and activity, we examined in vitro COX-2 and iNOS expression, as well as nitric oxide (NO) and prostaglandin E_2_ (PGE_2_) production. Both cell lines were pre-treated with increasing concentrations of the HA/B3 formulation for 2 h at 37 °C. Subsequently, Raw 264.7 macrophages were stimulated with 50 ng/mL lipopolysaccharide (LPS), and C28/I2 chondrocytes were stimulated with 1 ng/mL interleukin-1β (IL-1β). Following a 24 h incubation period, the culture medium and cell lysates were collected. NO and PGE_2_ levels in the culture medium were quantified using the adapted NCL ITA-7 method and an enzyme-linked immunosorbent assay (ELISA), respectively [41]. Cell lysates, prepared using Laemmli buffer, were used for semi-quantitative assessment of iNOS and COX-2 protein expression via Western blot analysis. As illustrated in Figure 2, treatment of Raw 264.7 macrophages with high concentrations of HA/B3 (i.e., ≥ 100/66.6) resulted in a significant decrease in PGE_2_ levels (Figure 2A), NO production (Figure 2E), and COX-2 (Figure 2C) and iNOS (Figure 2G) protein expression.

In contrast, treatment with HA alone did not elicit significant differences in NO or PGE_2_ levels, or COX-2 and iNOS protein expression, compared to the positive control (LPS-stimulated cells; Figure 2).

**Figure 3:**
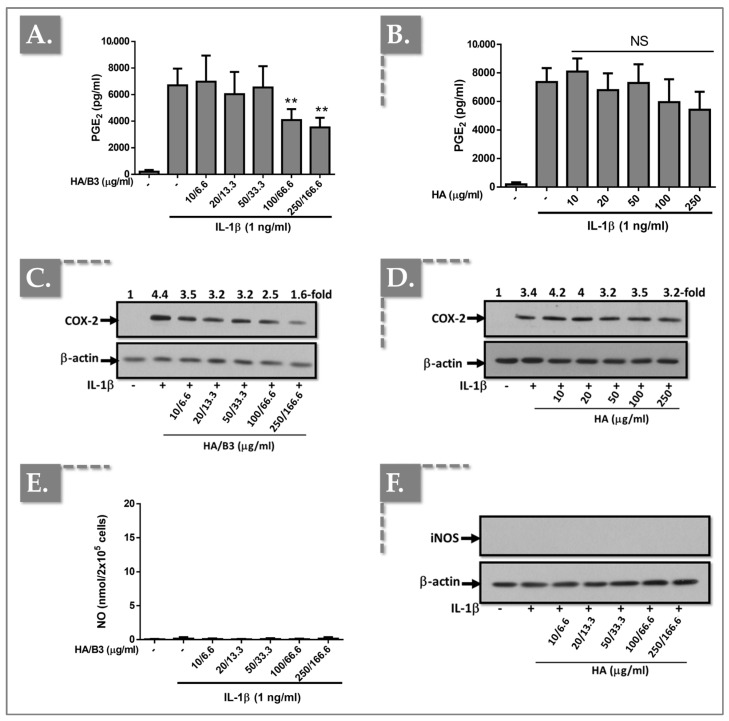
Investigation of the anti-inflammatory effects of the HA/B3 combination in C28/I2 chondrocytes. The cells were pretreated with increasing concentrations of HA alone or HA/B3 for 2 h. After this period, they were induced with 1 ng/mL IL-1β. They were then incubated for 24 h at 37 °C under 5% CO_2_ in a humidified environment. Then, 50 μL of culture medium was isolated and used for PGE_2_ (**A**,**B**) and NO assays (**E**). Samples were diluted 20-fold for the PGE_2_ assay. Volumes of 15 μL of cell lysate were loaded onto SDS-PAGE polyacrylamide gels, followed by transfer to nitrocellulose membranes. COX-2 (**C**,**D**) and iNOS (**F**) proteins were revealed by immunoblotting and detected with Rochester photosensitive film. Statistically significant differences (i.e., ** or *p*-value < 0.01) are outlined between the groups. Data are expressed as the mean of three independent tests (N = 3) and analyzed by one-way ANOVA (i.e., with a post hoc Tukey test). HA, hyaluronic acid; LPS, lipopolysaccharide; NO, nitric oxide. - not added, + treated, NS Not Significant.

In C28/I2 chondrocytes, treatment with HA/B3 at high doses (i.e., ≥100/66.6) resulted in a reduction in PGE_2_ production (Figure 3A) and COX-2 protein expression (Figure 3C).

Conversely, treatment with HA alone elicited a modest inhibition of IL-1β-induced PGE_2_ release (Figure 3B) and COX-2 expression (Figure 3D). Notably, NO release (Figure 3E) and iNOS protein expression (Figure 3F) were undetectable in the culture medium and cell lysates of C28/I2 chondrocytes, respectively.

### 3.3. HA/B3 Combinations Inhibit the Production of TNF-α in LPS-Induced Macrophages

**Figure 4:**
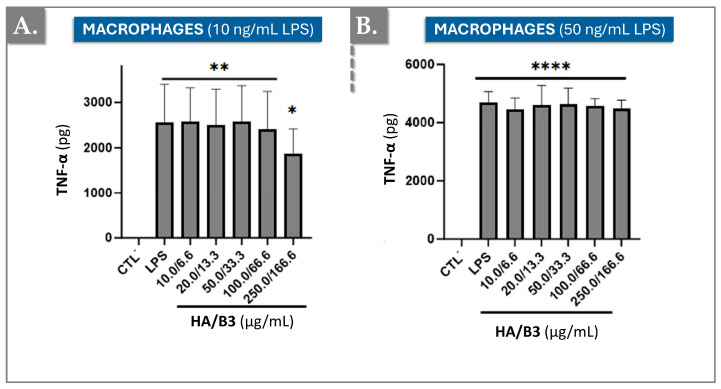
Levels of TNF-α induced by 10 ng/mL LPS (**A**) or by 50 ng/mL LPS (**B**) in Raw 264.7 macrophages exposed to different concentrations of HA/B3. The cells were pretreated with increasing concentrations of the HA/B3 combination for 2 h. After this period, they were induced with LPS and incubated for 24 h. Volumes of 50 μL of culture medium were isolated to quantify TNF-α by ELISA. Statistically significant differences (i.e., **** or *p*-value < 0.0001; ** or *p*-value < 0.01; * or *p*-value < 0.05) are outlined between the groups. Data are expressed as the mean of three independent tests (N = 3) and analyzed by one-way ANOVA (i.e., with a post hoc Tukey test). ELISA, enzyme-linked immunosorbent assay; HA, hyaluronic acid; LPS, lipopolysaccharide; TNF, tumour necrosis factor.

To further elucidate the anti-inflammatory effects of the HA/B3 combination, we investigated tumor necrosis factor-alpha (TNF-α) cytokine production in Raw 264.7 macrophages. Cells were pre-treated with increasing concentrations of the test formulation for 2 h and subsequently stimulated with 10 or 50 ng/mL LPS. Following a 24 h incubation period, the culture media were collected, and 50 μL aliquots (diluted 50-fold) were used to quantify TNF-α cytokine levels using an enzyme-linked immunosorbent assay (ELISA). Figure 4 presents the TNF-α levels observed for each treatment group.

The HA/B3 combination significantly reduced TNF-α production in cells stimulated with the low dose of 10 ng/mL LPS (Figure 4A). However, no effect was observed in RAW 264.7 macrophages stimulated with the higher dose of 50 ng/mL LPS, where the HA/B3 combination did not significantly alter TNF-α production (Figure 4B). 

### 3.4. HA/B3 Combinations Inhibit the MAP Kinase Signaling Pathway and Downregulate the Production of MMP-13

**Figure 5:**
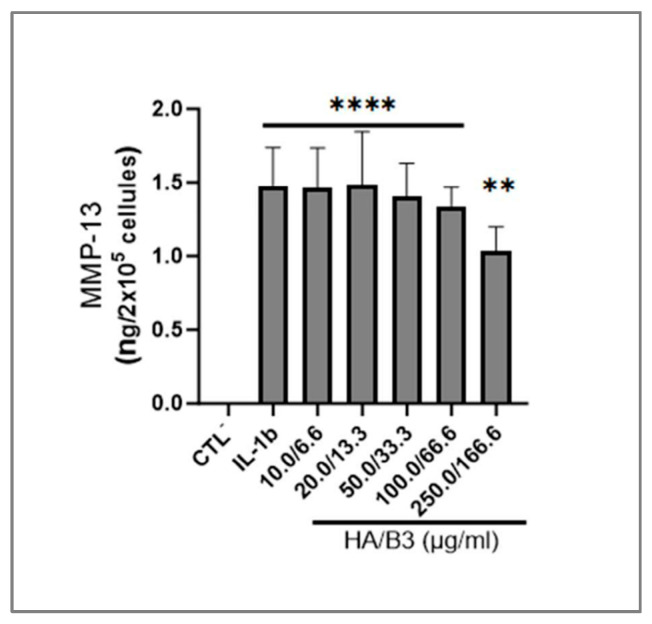
Investigation of the expression of MMP-13 in C28/I2 chondrocytes. The cells were pretreated with increasing concentrations of HA/B3 for 2 h. They were induced with 1 ng/mL IL-1β. After 1 h incubation at 37 °C, volumes of 20 μL of treated cell culture medium (i.e., 10-fold dilution) were used for ELISA quantification of MMP-13. Statistically significant differences (i.e., **** or *p*-value < 0.0001; ** or *p*-value < 0.01) are outlined between the groups. Data are expressed as the mean of three independent tests and analyzed by one-way ANOVA (i.e., with a post hoc Tukey test). ELISA, enzyme-linked immunosorbent assay; HA, hyaluronic acid; IL, interleukin; MMP, matrix metalloproteinase.

To evaluate the impact of the HA/B3 combination on metabolic pathways and enzymes involved in ECM degradation, we analyzed the expression of matrix metalloproteinase-13 (MMP-13) in C28/I2 chondrocytes. Specifically, MMP-13 levels were quantified in the culture medium after a 24 h incubation period. As depicted in Figure 5, high concentrations of HA/B3 effectively reduced IL-1β-induced MMP-13 production, indicating its anti-catabolic activity in these cells.

**Figure 6:**
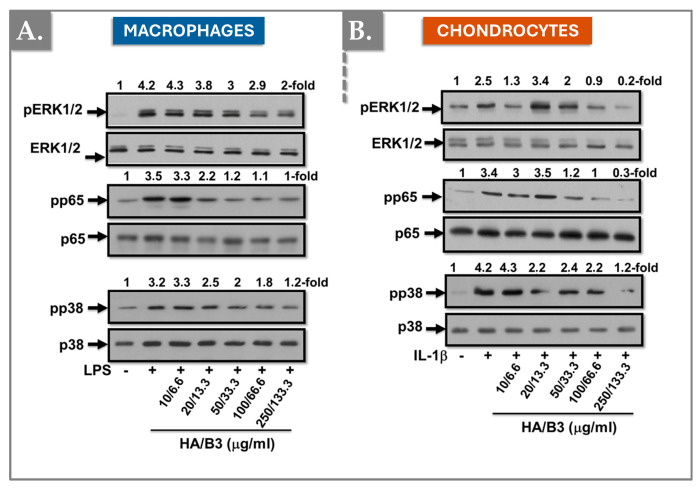
Investigation of the regulation of the MAP kinase signaling pathway in Raw 264.7 macrophages (**A**) and C28/I2 chondrocytes (**B**). The cells were pretreated with increasing concentrations of HA/B3 for 2 h. They were induced with 50 ng/mL LPS for macrophages and 1 ng/mL IL-1β for chondrocytes. After 1 h incubation at 37 °C, volumes of 15 μL culture medium were loaded for Western blot immunoblotting. HA, hyaluronic acid; IL, interleukin; LPS, lipopolysaccharide. - not added, + treated.

To further investigate the specific signaling pathways implicated in catabolic and inflammatory responses, Raw 264.7 macrophages and C28/I2 chondrocytes were pre-treated with increasing concentrations of HA/B3 for 2 h. Subsequently, macrophages were stimulated with 50 ng/mL LPS, and chondrocytes were stimulated with 1 ng/mL IL-1β. Cells were then incubated for 1 h at 37 °C in a humidified atmosphere containing 5% CO_2_. Cell lysates were collected using Laemmli buffer, and 15 μL samples were used to determine mitogen-activated protein kinase (MAPK) content. Figure 6 demonstrates that the HA/B3 combination effectively downregulated the active phosphorylated forms of ERK1/2 and p38 MAPK, as well as p65/NF-κB, in both cell lines.

Collectively, these data suggested that the HA/B3 combination downregulates the MAP kinase and NF-kB signaling pathways, producing a direct impact on catabolic and pro-inflammatory gene expression (Figure 5 and Figure 6).

### 3.5. HA/B3 Combinations Display Anti-Hypertrophic Effects in Rat Primary Chondrocytes

**Figure 7:**
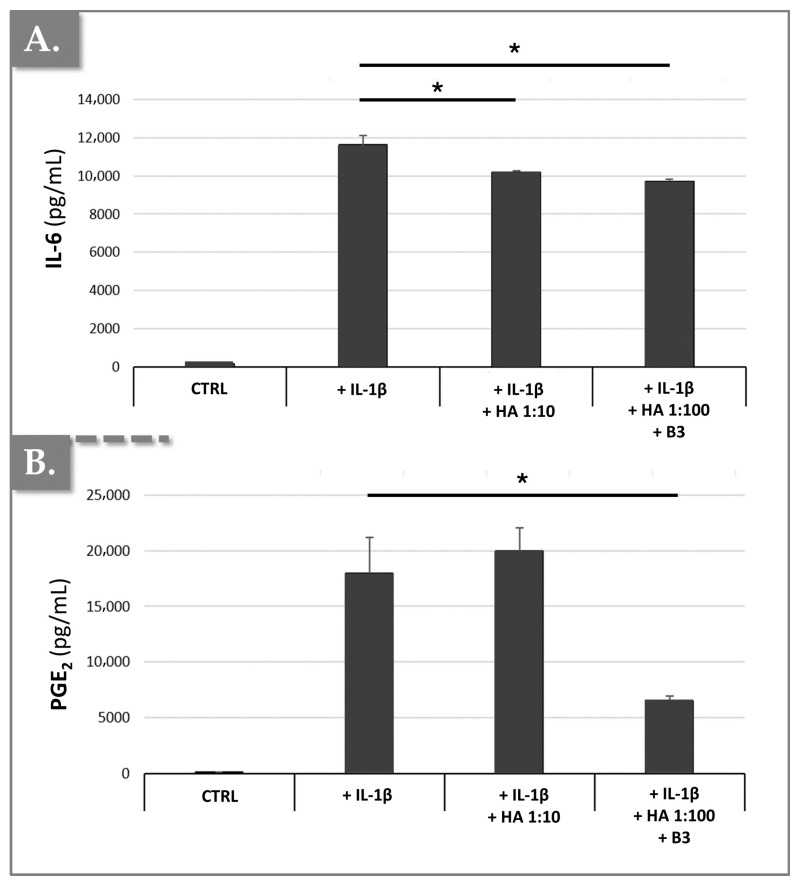
Effects of HA/B3 combinations on IL-1β-mediated hypertrophic-like changes in rat chondrocytes, assessed by soluble cytokine quantification. Rat chondrocytes were incubated with or without test compounds along with IL-1β. Quantities of IL-6 (**A**) and PGE_2_ (**B**) released into the culture medium were determined using ELISA analysis. Results (N = 3) are expressed as means with standard deviations as error bars. Statistically significant differences (i.e., * or *p*-value < 0.05) were evidenced between the groups (i.e., with a Mann–Whitney test). HA, hyaluronic acid.

To induce chondrocyte hypertrophic-like changes, mimicking those observed in osteoarthritis (OA), such as the suppression of type II collagen and aggrecan expression, and the induction of MMP-13, IL-6, and PGE_2_ production, primary rat chondrocytes were treated with IL-1β for three days. Subsequently, the cells were incubated with or without the HA/B3 combination, in the continued presence of 10 ng/mL IL-1β. Following a 72 h treatment period, culture medium samples were collected to quantify IL-6 and PGE_2_ production using enzyme-linked immunosorbent assays (ELISA). Additionally, RNA was extracted to determine the expression of type II collagen, aggrecan, and MMP-13 using reverse transcription-quantitative polymerase chain reaction (RT-qPCR) analysis. As expected, and illustrated in Figure 7, IL-1β treatment resulted in a significant increase in both IL-6 and PGE_2_ production.

The results demonstrated that IL-1β-induced IL-6 production was significantly inhibited by 16% in primary chondrocytes treated with a 1:100 dilution of HA/B3 (i.e., 25/250 μg/mL HA/B3, Figure 7A). Furthermore, this treatment resulted in a 63% inhibition of IL-1β-induced PGE_2_ production (Figure 7B). Notably, a comparable inhibition of IL-6 production, but no significant effect on PGE_2_ production, was observed when chondrocytes were treated with a 1:10 dilution of HA alone (i.e., lacking niacinamide; Figure 7). These findings suggested that the HA/B3 formulation effectively counteracts the detrimental inflammatory effects mediated by IL-1β in rat chondrocytes. If we consider that HA deploys negligible effects and the main anti-inflammatory action derives from B3, the fact that a slight reduction in IL-6 and PGE_2_ was recorded with 250 μg/mL B3 (i.e., after 10 ng/mL IL-1 β stimulation) is interesting. Notably, the results presented above showed a 60% reduction in PGE_2_ levels in treated macrophages and in treated chondrocytes. In the case of primary rat chondrocytes, a 63% inhibition of PGE_2_ was noted, even with varying IL-1β concentrations. Thus, it may be set forth that B3 seems to affect the COX-2/PGE_2_ pathway, in particular.

**Figure 8:**
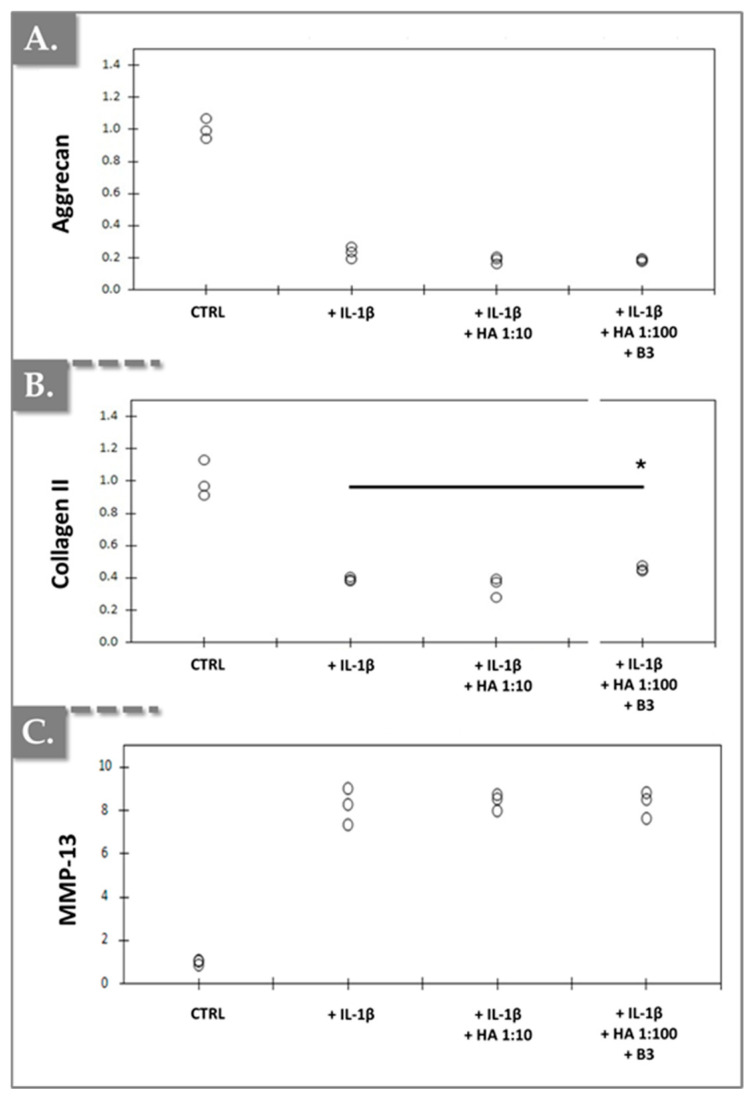
Effects of HA/B3 combinations on IL-1β-mediated hypertrophic-like changes in rat chondrocytes (N = 3), assessed by chondrogenic gene expression analysis. Rat chondrocytes were incubated with or without test compounds along with IL-1β. Relative normalized expression levels of aggrecan (**A**), type II collagen (**B**), and MMP-13 (**C**) were determined using RT-qPCR analysis. Expression levels were normalized against reference genes (i.e., β-actin and RPL19) and compared to expression levels in IL-1β-treated chondrocytes. Statistically significant differences (i.e., * or *p*-value < 0.05) were evidenced between the groups (i.e., with a Mann–Whitney test). HA, hyaluronic acid.

Additionally, as anticipated, IL-1β treatment resulted in a slight decrease in the expression of aggrecan and type II collagen. However, an unexpected increase in MMP-13 expression was observed (Figure 8).

A modest, yet statistically significant, increase in type II collagen expression was observed in chondrocytes treated with a 1:100 dilution of HA/B3 compared to cells treated with IL-1β alone (Figure 8B). However, no significant alterations in aggrecan or MMP-13 expression were detected in the other treatment groups, at the tested dilutions, compared to the IL-1β-treated cells (Figure 8). Furthermore, treatment of primary chondrocytes with a 1:10 dilution of HA alone (lacking niacinamide) did not elicit any significant effects on the expression of these three markers compared to IL-1β-treated cells. Collectively, the in vitro data obtained from primary rat chondrocytes corroborated that the incorporation of niacinamide into HA-based hydrogels conferred anti-inflammatory and, to some extent, indications of a potential anabolic response (Figure 6, Figure 7 and Figure 8).

### 3.6. General Discussion

Hyaluronic acid (HA) is renowned for its unique viscoelastic properties and high water-holding capacity. Beyond these mechanical attributes, HA exhibits a broad spectrum of anti-inflammatory, analgesic, and antioxidant activities [27,28]. In OA, a notable downregulation of critical ECM molecules, including HA and type II collagen, has been consistently observed in affected joints [1,3]. Intra-articular HA injections have emerged as a clinically viable approach for alleviating OA-related pain, with numerous studies demonstrating short- and medium-term benefits [42]. However, the long-term therapeutic efficacy of HA injections remains a subject of debate.

The high-molecular-weight HA polymer is susceptible to rapid degradation by free radicals and hyaluronidases, yielding lower molecular weight fragments that may lack the desired therapeutic effects. This molecular weight-dependent activity has prompted research efforts to stabilize HA through chemical modifications or the incorporation of antioxidant molecules capable of scavenging free radicals, thereby prolonging the therapeutic window [16,18,19,20]. The addition of antioxidant compounds not only enhances the stability of viscosupplement products within the syringe but also extends their post-administration efficacy [15,19]. As previously noted, exogenous HA exerts its effects primarily through interactions with ICAM-1, HMMR, and CD44 [31]. Furthermore, HA-binding proteins play a crucial role in transducing extracellular signals to the cytoplasm [32,33].

Our experimental data demonstrated that HA, at concentrations ranging from 10.0 to 500.0 μg/mL, exhibited no significant cytotoxicity in Raw 264.7 macrophages and SW1353 chondrocytes (Figure 1A,D). Cellular viability remained consistently above 90% for both cell lines. Similarly, niacinamide and the HA/B3 combination did not induce cytotoxicity in chondrocytes at the tested concentrations (Figure 1E,F). However, in Raw 264.7 macrophages, B3 and the HA/B3 combination showed a trend towards toxicity at higher concentrations, although the differences compared to untreated cells were not statistically significant (Figure 1B,C). A study demonstrated that niacinamide at high concentrations (i.e., ≥5 mM) induces accelerated mitophagy through upregulation of GAPDH and ATG12, selectively eliminating mitochondria with low membrane potential and thereby lowering mitochondrial mass [43]. This mechanism could similarly explain the apparent decline in RAW 264.7 metabolic activity at higher B3 doses.

To further investigate the anti-inflammatory properties of the HA/B3 combination, we quantified NO and PGE_2_ levels and assessed the expression of COX-2 and iNOS. The HA/B3 combination effectively downregulated COX-2 and iNOS expression in Raw 264.7 macrophages (Figure 2C,G). However, no direct influence of HA on NO and PGE_2_ levels was observed (Figure 2A,E). Notably, NO and PGE_2_ are key mediators of the inflammatory process. HA is known to suppress COX-2 and iNOS mRNA expression via its CD44 receptor, thereby reducing PGE_2_ and NO production [44,45]. Moreover, other studies suggest that one of the pathways leading to iNOS inhibition involves poly(ADP-ribose) polymerase (PARP). Overall, the inhibition of iNOS is a complex process mediated by different mechanisms. From this standpoint, niacinamide has already been shown to interfere with iNOS indirectly through multiple pathways [46,47]. Our findings suggest that the metabolic pathways governing NO and PGE_2_ production may not be exclusively COX-2/iNOS-dependent and could involve additional mechanisms [48,49,50]. In chondrocytes, iNOS was undetectable, and the HA/B3 formulation did not significantly affect COX-2 expression (Figure 3C,F). Nevertheless, a trend toward a dose-dependent decrease in PGE_2_ levels was observed (Figure 3A). Although the effect was not statistically significant compared to the positive control, these results point to a possible anti-inflammatory effect of HA/B3 in both Raw 264.7 and C28/I2 cells. Specifically, it downregulated enzymes involved in NO and PGE_2_ production in macrophages and also reduced PGE_2_ production in chondrocytes at higher concentrations (Figure 2 and Figure 3). B3 has previously been reported to influence nitric oxide synthase (NOS) activity, although the underlying mechanisms remain to be clarified [51,52]. At the redox level, nicinamide exhibits a concentration-dependent dual behavior. At moderate doses, it stabilizes redox balance, whereas at higher concentrations it induces ROS accumulation, lipid peroxidation, and GSH depletion, leading to transient oxidative stress and mitochondrial depolarization [43,53]. This biphasic effect reflects a “ROS-induced ROS release” process in which excessive ROS trigger mitochondrial dysfunction and apoptosis [53]. Such findings indicate that the cellular response to B_3_ depends on dose and oxidative context. Within the HA/B3 system, this redox modulation may fine-tune inflammatory signaling, where moderate ROS activate adaptive defenses while HA limits excessive oxidative damage.

Interleukin-1β (IL-1β) and tumor necrosis factor-α (TNF-α) are pivotal pro-inflammatory cytokines implicated in OA pathogenesis, primarily produced by macrophages within articular cartilage. To evaluate the impact of the HA/B3 combination on cytokine production, we quantified IL-1β and TNF-α levels in treated macrophages. IL-1β was undetectable, possibly requiring higher stimulation levels for detection. The HA/B3 combination had a limited effect on TNF-α levels in macrophages stimulated with 50 ng/mL lipopolysaccharide (LPS) (Figure 4B). However, in cells stimulated with 10 ng/mL LPS, a 27% reduction in TNF-α levels was observed compared to the positive control (Figure 4A). These findings suggest that the high inflammatory burden induced by 50 ng/mL LPS may have obscured the anti-inflammatory effects of the HA/B3 formulation (Figure 4). It should be mentioned that, while it was not possible to observe the benefits of HA/B3 in TNF-α reduction, it was possible to show beneficial effects, notably for COX and NO, at the same concentration (50 ng/mL LPS). Future investigations will aim to quantify COX-2 and iNOS expression using sensitive ELISA-based assays rather than Western blotting, in order to improve accuracy and reproducibility. Although a complete dose–response validation was not performed, the present data indicate that HA/B3 may exert anti-TNF-α effects more clearly under moderate inflammatory conditions, which could be particularly relevant to early rather than advanced stages of OA. 

Mitogen-activated protein kinases (MAPKs) play a critical role in chondrocyte physiology, regulating processes such as differentiation, senescence, and ECM production [54]. The NF-κB (p65) signaling pathway, among others, induces the expression of matrix metalloproteinase-13 (MMP-13), a key enzyme responsible for ECM degradation in articular cartilage [55,56,57]. To assess the effects of the HA/B3 combination on MAPK signaling and MMP-13 expression, we examined the phosphorylation status of p38, NF-κB/p65, and ERK1/2. In macrophages, the HA/B3 combination downregulated the phosphorylated, active forms of p38 and ERK/p65 (Figure 6A). In chondrocytes, all three active kinases were downregulated in a dose-dependent manner (Figure 6B). These results are consistent with previous reports demonstrating that high molecular weight HA can suppress ERK signaling through its receptors RHAMM/CD168 and CD44 [36,58]. Similarly, the HA/B3 combination reduced MMP-13 protein levels by 30% compared to the control group (Figure 5). However, this effect was not replicated at the gene expression level in rat chondrocytes, possibly due to the tenfold higher IL-1β concentration used in that assay and the differences in experimental methods (ELISA versus PCR, Figure 5 and Figure 8C). Of note, discrepancies between protein and gene expression levels may arise from variations in protein clearance or availability. Overall, the primary rat chondrocyte assays highlighted the importance of carefully selecting test and challenge item concentrations to elicit significant biological effects.

Clinically, the HA/B3 combination holds significant promise as a multifaceted therapeutic approach for OA. Vitamin B3 may act as a salt, with possible hydrogen bonding with the hydrophilic groups of the HA backbone [59]. Its ability to mitigate inflammation, modulate catabolic enzymes, and protect cartilage integrity positions it as a promising candidate for integration into existing OA management strategies. The targeted anti-inflammatory effects, particularly the reduction in PGE_2_ levels, are crucial in OA, where elevated PGE_2_ contributes to cartilage degradation, pain, and disease progression [60]. The HA/B3 combination’s ability to inhibit TNF-α production in macrophages, particularly in mild to moderate inflammation, suggests that it may be most effective in early-stage OA or during periods of lower inflammatory activity [61]. Furthermore, its role in modulating MMP-13 levels indicates that it could potentially preserve cartilage integrity and slow disease progression [62].

### 3.7. Study Limitations and Perspectives

This study, while providing valuable insights into the functional attributes of HA/niacinamide (HA/B3) combinations, acknowledges several limitations that warrant consideration. Firstly, the reliance on immortalized cell lines (Raw 264.7 macrophages and C28/I2 chondrocytes) and primary rat chondrocytes restricts the direct translatability of our findings to human physiological conditions. As such, future investigations employing human primary chondrocytes and synoviocytes would significantly enhance the clinical relevance of our observations. Specifically, given that our study demonstrated significant anti-inflammatory effects of HA/B3 in both macrophage and chondrocyte cell lines, including the reduction in key inflammatory mediators like PGE_2_ and TNF-α, validating these findings in human primary cells would strengthen their potential clinical application. Additionally, the interpretation of NO and PGE_2_ reduction remains limited, as no direct correlation analysis between mediator levels and COX-2/iNOS expression was performed. Future studies should include statistical correlation with downstream mediators and a quantified statistical total protein ratio to confirm pathway involvement. Furthermore, the study design did not incorporate a three-dimensional (3D) co-culture or organoid-like system that could reproduce the native interactions between macrophages and chondrocytes within the osteoarthritic joint. Advanced 3D co-culture models have shown that activated macrophages amplify catabolic and inflammatory responses in chondrocytes, while non-activated macrophages exert protective effects. Integrating HA/B3 into such models in future work would provide valuable insight into its ability to modulate macrophage–chondrocyte crosstalk and maintain joint microenvironment homeostasis [63]. However, the literature is consistent with these elements, given the reported reduction in NO/iNOS levels by niacinamide.

Secondly, although the MTS assay revealed that cellular viability remained above 80% across all treatment groups, a more comprehensive assessment of cytotoxicity would have benefited from the inclusion of lactate dehydrogenase (LDH) release measurements using OCDE-recommended assay durations (48–72 h). LDH release is a well-established indicator of cellular membrane integrity and damage, providing complementary information to the metabolic activity assessed by the MTS assay. Integrating LDH data would offer a more robust evaluation of the potential toxicological effects of HA, niacinamide, and their combinations, particularly in light of the observed trend towards reduced viability in Raw 264.7 macrophages at higher niacinamide concentrations.

Furthermore, the functional data obtained for HA/B3 combinations could be enriched by comparative analyses with alternative formulations. Specifically, exploring the effects of HA hydrogels incorporating other antioxidant compounds would provide a broader perspective on the optimal product composition for therapeutic applications. Given the observed anti-inflammatory and anti-catabolic properties of HA/B3, particularly in reducing MMP-13 and MAPK signaling, comparing these effects with other antioxidant-modified HA hydrogels could help to identify superior formulations for OA treatment.

Finally, while our study elucidated the anti-inflammatory and anti-catabolic properties of HA/B3 combinations, a more in-depth investigation of their anabolic effects is warranted. For instance, while we observed a slight increase in type II collagen expression in rat chondrocytes treated with HA/B3, a more comprehensive analysis of ECM synthesis and remodeling, including the expression of aggrecan, immunostaining, and other cartilage-specific markers, would provide a more complete understanding of the regenerative potential of these hydrogels. Additionally, examining the long-term effects of HA/B3 on chondrocyte phenotype (e.g., maintenance of the differentiated chondrocyte state) and ECM homeostasis would be valuable. Moreover, assessing the impact of HA/B3 on other anabolic markers, like SOX9, would be extremely useful. Such complementary preclinical studies would provide a more holistic view of the therapeutic potential of HA/B3 in promoting cartilage repair and regeneration in OA joints.

## 4. Conclusions

In conclusion, this study provides preliminary insights into the multifaceted anti-arthritic effects of the HA/B3 combination, offering a preliminary rationale for its potential clinical application in OA management. Notably, in vitro assessments demonstrated that both HA/B3 and its individual components exhibited low cytotoxicity in Raw 264.7 macrophages and SW1353 chondrocytes, confirming their biocompatibility at the tested concentrations.

Furthermore, we observed that HA/B3 was able to downregulate iNOS and COX-2 expression in LPS-stimulated macrophages and IL-1β-treated chondrocytes, suggesting an inhibitory effect on key enzymes involved in inflammatory mediator production. However, while enzyme expression was reduced, the corresponding reductions in NO and PGE_2_ production were minimal, indicating potential involvement of alternative pathways in their synthesis. In C28/I2 chondrocytes, iNOS and NO were undetectable, and COX-2 expression remained in part unaffected by HA/B3. Nonetheless, a dose-dependent trend toward reduction in PGE_2_ levels was observed, highlighting a possible inhibitory effect of HA/B3 on PGE_2_ production in these cells.

The HA/B3 combination also demonstrated a capacity to inhibit TNF-α production in RAW 264.7 macrophages, particularly under conditions of moderate LPS-induced inflammation, but no effect was observed under stronger inflammatory stimulation (i.e., 50 ng/mL LPS). This observation suggests that HA/B3 may be most effective in mitigating inflammatory responses in the early stages of OA or during periods of lower inflammatory activity, as higher LPS or IL-1β concentrations appeared to attenuate the observed effects.

Moreover, our study provided evidence that HA/B3 can effectively modulate MMP-13 levels, a crucial enzyme in cartilage ECM degradation. Specifically, HA/B3 treatment led to a significant reduction in MMP-13 protein levels in human chondrocytes, indicating its potential to preserve cartilage integrity by inhibiting catabolic processes. The observed anti-inflammatory and anti-catabolic properties, coupled with the demonstrated biocompatibility, position HA/B3 as a promising therapeutic strategy for mitigating OA-related inflammation and cartilage degradation.

## Figures and Tables

**Table 1:** Primer sequences for qPCR assays.

Gene	Forward Primer Sequence	Reverse Primer Sequence
Type II collagen	TCCCTCTGGTTCTGATGGTC	CTCTGTCTCCAGATGCACCA
Aggrecan	ACACCCCTACCCTTGCTTCT	AAAGTGTCCAAGGCATCCAC
MMP-13	CTGACCTGGGATTTCCAAAA	ACACGTGGTTCCCTGAGAAG
RPL19	TGCCGGAAGAACACCTTG	GCAGGATCCTCATCCTTCG
β-actin	CCAACCGTGAAAAGATGACC	ACCAGAGGCATACAGGGACA

## Data Availability

The original contributions presented in the study are included in the article, further inquiries can be directed to the corresponding author.

## Abbreviations

The following abbreviations are used in this manuscript:

DMEM	Dulbecco’s modified Eagle medium
ECM	extracellular matrix
ELISA	enzyme-linked immunosorbent assay
FBS	fetal bovine serum
FDA	US Food and Drug Administration
H_2_O_2_	hydrogen peroxide
HA	hyaluronic acid
HYAL	hyaluronidases
IL	interleukins
LPS	lipopolysaccharide
MDa	megadalton
min	minute
NA	non-applicable
NO	nitric oxide
OA	osteoarthritis
OD	optic density
PBS	phosphate-buffered saline
PRP	platelet-rich plasma
RNA	ribonucleic acid
ROS	reactive oxygen species
TNF	tumor necrosis factor
USA	United States of America
